# Endothelial dysfunction in acute brain injury and the development of cerebral ischemia

**DOI:** 10.1186/cc14523

**Published:** 2015-03-16

**Authors:** S Van Ierssel, VM Conraads, EM Van Craenenbroeck, Y Liu, AI Maas, PM Parizel, VY Hoymans, CJ Vrints, PG Jorens

**Affiliations:** 1 University Hospital Antwerp, Edegem, Belgium; 2 The Third Xiangya Hospital, Changsha, China

## Introduction

Cerebral ischemia (CeI) is a major complicating event after acute brain injury (ABI) in which endothelial dysfunction is a key player.

## Methods

We studied cellular markers of endothelial dysfunction and peripheral reactive hyperemia index (RHI) in 26 patients with ABI at admission and after 6 and 12 days, and compared these with healthy volunteers (*n *= 15). CeI was determined clinically or using computer tomography.

## Results

**Figure 1:**
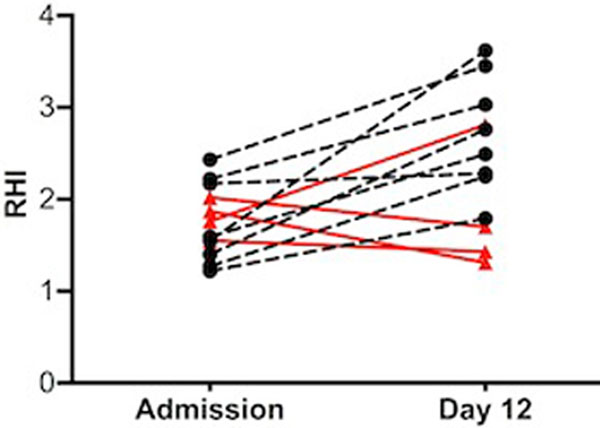


In patients with ABI, RHI at admission was significantly reduced compared with healthy subjects (*P *= 0.003), coinciding with a decrease in circulating endothelial progenitor cells (EPC) (*P *= 0.002) (Table 1). The RHI recovered in eight patients without development of CeI (Figure 1, black), but failed to fully recover by day 12 in three out of four patients that developed CeI (Figure 1, red). Despite recovery of the RHI within 12 days in these patients (*P *= 0.003), the EPC count remained significantly lower after 12 days in patients with ABI (*P *= 0.022) (Table 1). CD31^+ ^T cells and endothelial microparticles were not different between controls and patients. No differences were noted in cellular markers of endothelial dysfunction in patients developing CeI and those not.

**Table 1:** Evolution in time of markers of endothelial dysfunction after acute brain injury.

	Healthy volunteers			
	**(*n *= 15) **	**D0**	**D6**	**D12**

EPC/10^5^ PBMC	24.1 ± 6.3	11.9 ± 2.2	11.0 ± 1.8	12.6 ± 2.3
%CD31^+^ of T cells	43.1 ± 2.6	42.4 ± 2.4	40.1 ± 3.0	43.4 ± 2.8
RHI (*n *= 12)	2.41 ± 0.14	1.68 ± 0.12	2.14 ± 0.15	2.46 ± 0.21

## Conclusion

Patients with ABI exhibit impaired microvascular endothelial function measured as RHI and a decreased circulating level of EPC.

